# Measuring Hemoglobin Levels in the Optic Disc of Parkinson's Disease Patients Using New Colorimetric Analysis Software

**DOI:** 10.1155/2014/946540

**Published:** 2014-12-23

**Authors:** Maria Pilar Bambo, Elena Garcia-Martin, Maria Satue, Susana Perez-Olivan, Silvia Alayon, Marta Gonzalez-Hernandez, Vicente Polo, Jose Manuel Larrosa, Manuel Gonzalez-De la Rosa

**Affiliations:** ^1^Ophthalmology Department, Miguel Servet University Hospital, Consultas Externas de Oftalmologia, C/Padre Arrupe, 50009 Zaragoza, Spain; ^2^Aragones Institute of Health Sciences (IIS Aragon), 50009 Zaragoza, Spain; ^3^Systems Engineering Department, University of La Laguna, 38071 La Laguna, Spain; ^4^Ophthalmology Department, University of La Laguna, 38071 La Laguna, Spain

## Abstract

*Objective.* To evaluate a new method of measuring hemoglobin (Hb) levels and quantifying the color changes in the optic nerve head of Parkinson's disease (PD) patients. We also compared differences in retinal nerve fiber layer (RNFL) thicknesses obtained using spectral domain optical coherence tomography (OCT) device between PD group and healthy group. *Methods.* One hundred and fifty-five PD patients and 91 sex- and age-matched healthy subjects were included in this cross-sectional study. OCT examinations and one photograph of the optic disc were performed. The Laguna ONhE (“optic nerve hemoglobin”; Insoft SL, Tenerife, Spain) software was used to analyze the Hb level on the acquired optic disc photographs. *Results.* PD patients exhibited significantly reduced mean optic disc Hb percentages (57.56% in PD, 67.63% in healthy subjects; *P* = 0.001) as well as reduced Hb in almost all analyzed sectors, with the largest differences detected in the inferior and nasal sectors. RNFL parameters were significantly reduced in PD patients compared with healthy subjects, especially in the inferior quadrant. *Conclusions.* Measurements of optic disc Hb levels obtained with the Laguna ONhE software had good ability to detect optic nerve color changes (more papillary paleness and consequently this could suggest optic atrophy and axonal loss) in PD patients.

## 1. Introduction

Parkinson's disease (PD) is a neurodegenerative process that leads to a selective loss of dopaminergic neurons, mainly in the basal ganglia of the brain [1]. Neurons and neural circuits outside the basal ganglia can be affected simultaneously or even before there are noticeable changes in the substantia nigra [2]. The visual system is also altered in PD, especially the visual field corresponding to the fovea [3].

Retinal ganglion cell loss can be detected using ocular imaging technologies such as optical coherence tomography (OCT) [4], which provide noninvasive, rapid, objective, and reproducible measurements of the retinal nerve fiber layer (RNFL). Spectral-domain OCT in previous studies of PD patients demonstrated a reduced macular thickness [5–9] and RNFL thickness [9–11] in PD patients compared with healthy controls.

Axonal loss in the optic nerve is also observed as a progressive pallor by fundus examination with an ophthalmoscope. The human eye, however, cannot quantify the axonal loss or detect early axonal loss (papillary atrophy can only be observed when more than 50% of nerve fibers have been lost) [12]. The Laguna ONhE (optic nerve head hemoglobin) program is a new method designed by a group of ophthalmologists and engineers that allows for the measurement of hemoglobin levels (Hb) at the optic nerve head using conventional fundus color photographs that compensate for different variables, such as illumination or lens absorption and diffusion. The Laguna ONhE has already been used for diagnosis of glaucomatous optic neuropathy, demonstrating similar diagnostic ability compared with classic functional and structural tests used in glaucoma [13], and this new technology has also proved useful for analyzing axonal loss in neuroophthalmologic patients, such as those with multiple sclerosis [14].

Unlike other regions of the posterior pole of the eye, the optic nerve head contains a significant amount of just one pigment, Hb, which is responsible for its particular color. Therefore, measuring the amount of Hb in different sectors of the optic nerve head can be used to quantify the color changes that occur in the disc as a result of different processes, such as neurodegenerative disease.

Given the value of RNFL and macular thicknesses measurements as a method of evaluating and diagnosing PD [5–11], the aim of the present study was to investigate differences in the optic nerve color between a group of patients with PD and a group of healthy subjects, using Hb as a reference pigment. Our study also analysed the differences in RNFL thicknesses between the two groups obtained using two spectral domain OCT devices: the Cirrus High Definition (HD) OCT and the Spectralis OCT. To the best of our knowledge, the ability of the Laguna ONhE to detect changes in Hb levels in different sectors of the papilla (and therefore papillary color changes) has not been examined in patients with PD compared with healthy subjects.

## 2. Materials and Methods

The design of the study followed the Declaration of Helsinki Principles and the study protocol was approved by the Clinical Research Ethics Committee of Aragon (Zaragoza, Spain). Informed written consent was obtained from all participants.

### 2.1. Subjects and Measurement Protocol

Required inclusion criteria were best-corrected visual acuity (BCVA) of 20/40 or better, refractive error within ±5.00 diopters equivalent sphere and ±2.00 diopters astigmatism, and transparent ocular media (nuclear color/opalescence, cortical or posterior subcapsular lens opacity <1), according to Lens Opacities Classification System III [15]. Exclusion criteria included previous intraocular surgery, diabetes, or other diseases affecting the visual field or neurologic system and current use of medications that could affect visual function. Exclusion criteria also included glaucoma signs (applanation intraocular pressure over 20 mm Hg), previous intraocular surgery, cup-to-disc ratio of 0.5 or higher, or arcuate nerve fiber bundle visual field defects. Patients with severe PD were not included due to their inability to complete the exploratory protocol.

One hundred and fifty-five patients with PD and 91 sex- and age-matched healthy subjects were included in the study from January 2013 to September 2013. Diagnosis of PD was based on the UK Brain Bank Criteria, which included bradykinesia and one additional symptom, that is, rigidity, 4–6 Hz resting tremor, or postural instability [16, 17]. For diagnosis of “definite” PD, three or more of the following criteria were required in combination with those mentioned above: unilateral onset, progressive disorder, persistent asymmetry affecting the side of onset the most, excellent response (70%–100%) to levodopa, severe levodopa-induced chorea, levodopa response for 5 years or more, and clinical course of 10 years or more [17, 18].

All subjects underwent a complete neuroophthalmologic examination, which included assessment of BCVA; color vision evaluation (using Ishihara's isochromatic charts); eye movement; pupillary, anterior segment, and fundoscopic examinations; Goldmann applanation tonometry; and OCT examinations using the Cirrus HD OCT (Carl Zeiss Meditec, Dublin) and the Spectralis OCT (Heidelberg, Engineering Inc., Heidelberg, Germany). One photograph of the optic disc was obtained using a Canon CF 60 DSi retinograph (Canon Incorporation, Tokyo, Japan) connected to a Canon EOS 1DS Mark III body camera. Each eye was considered separately and only one eye of each subject was included randomly in the analyses. Disease duration and severity according to different validated scales were also recorded.

### 2.2. OCT Evaluation

The OCT tests were performed to obtain measurements of the peripapillary RNFL using the Cirrus and Spectralis OCT devices, both of which were used in random order to prevent any effect of fatigue bias. The same experienced operator performed all scans. An internal fixation target was used because it provides the highest reproducibility [4]. Scan quality was assessed before the analysis, and poor-quality scans were rejected. Three patients were excluded because a centered scan could not be acquired due to poor fixation. Ten images with artifacts or missing parts, or showing seemingly distorted anatomy, were excluded [19]. To obtain good-quality and centered images, repeat scan acquisition using the Cirrus OCT device was required in 11 eyes and with the Spectralis OCT device in 8 eyes.

The Cirrus OCT optic disc protocol generates 200 × 200 voxel images from 200 linear scans that are performed by 200 A-scans. This option analyzes a 6-mm cube around the optic nerve. In each series of scans, mean RNFL thickness, quadrant RNFL thickness (superior, inferior, temporal, and nasal), and thickness at 12-clock-hours papillary sector of 30° RNFL were analyzed. The hour sectors were assigned a number from position H1 to H12 in the clockwise direction for the right eye and in the counterclockwise direction for the left eye. The RNFL Spectralis protocol generates a map with mean thickness and six sector thicknesses (superonasal, nasal, inferonasal, inferotemporal, temporal, and superotemporal in the clockwise direction for the right eye and counterclockwise for the left eye). RNFL acquisitions were obtained by the same observer using TruTrack eye-tracking technology that recognizes, locks onto, and follows the patient's retina during scanning and automatically places follow-up scans to ensure accurate monitoring of disease progression (we use this technology in our study in order to perform longitudinal studies in the future with this sample).

### 2.3. Optic Disc Photograph Evaluation

The Laguna ONhE program (Insoft SL, Tenerife, Spain) analyzed three spectral components of optic nerve head photographs: blue, green, and red. Optic nerve head areas with high Hb levels reflect mainly red light. In contrast, areas with a low Hb component reflect a lower proportion of the red component compared to the green and blue light. Using different concentrations or different thicknesses of various red blood cell dilutions, it may be established experimentally that the photographic images obtained with this technique can be used to determine the amount of Hb. Based on the reflected amounts of red, green, and blue light, in the photographic images of the cell dilutions, the results of several formulas were almost linearly proportional to the amount of Hb present [13].

**Figure 1:**
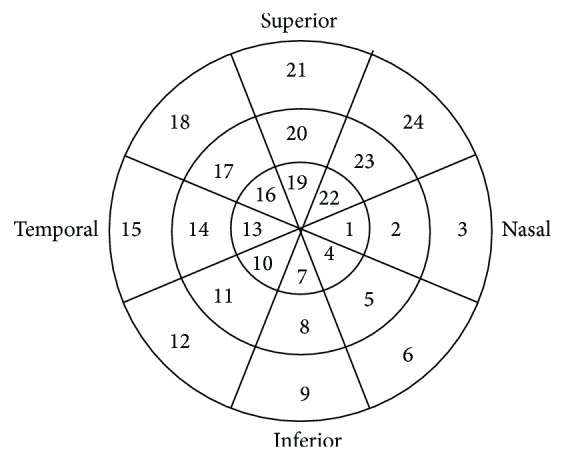
A papillary image was divided automatically by the Laguna ONhE (optic nerve head hemoglobin) device into eight 45° radial sectors and two concentric rings comprising 1/3 and 2/3 of the disc radius. The Laguna ONhE program analyzed the amount of hemoglobin in each of these 24 sectors and the average hemoglobin.

The Laguna ONhE software uses mathematical algorithms for automatic component segmentation to perform a semiautomatic delimitation of the optic nerve head border and to identify the central retinal vessels. Thus, two areas of the optic nerve head were defined: the central retinal vessels and the optic nerve head tissue itself. To obtain absolute and reproducible results from the papilla, a reference pattern is needed. The reference value must be obtained inside the eye and subject to the same variables (the intensity and spectral composition of the illuminating light, lens absorption, etc.). Because effects of lens deterioration (on vessels and on tissue) are proportional, when measuring the differences between the distances of green and blue at the corresponding pixels to vessels, the extent of this absorption-diffusion on the tissue may be estimated. So, the result obtained for the vessels was used as the reference value for calculating the Hb content in the tissue [20]. The image of the papilla was divided automatically into eight 45° radial sectors, and two concentric rings were also defined, comprising 1/3 and 2/3 of disc radius, obtaining average Hb and 24 sectors as shown in Figure 1. There are several factors that hinder the automatic analysis of the optic nerve head images: lighting issues, type of camera used, saturation level of the images, patient cooperation, and so forth. For this reason, the program has a preprocessing system of the images that prepares and improves retinal images for the subsequent segmentation stage. This preprocessing system prevents continued analysis of photographs that meet certain luminance and saturation criteria, and, in turn, allows for the use of images from different cameras. Finally, the influence of the lens status was compensated for by analyzing the differences between the green and blue components before calculating the Hb content. The blue, green, and red components were assessed using an image analysis program with the Matlab image processing toolbox (The MathWorks, Inc., Natick, Massachusetts).

### 2.4. Statistical Analysis

This was an observational, prospective cross-sectional study. All data analyses were performed using SPSS software version 20.0 (SPSS Incorporation, Chicago, Illinois) statistical software. The Kolmogorov-Smirnov test was used to assess sample distribution. Given the normal distribution of the data, the OCT and Hb parameters between healthy and PD patients were compared using Student's *t*-test with Bonferroni correction for multiple comparison, and correlations were examined by Pearson's test. Correlations between optic disc Hb percentages and disease severity (assessed by the Hoehn-Yahr stages) were also analyzed in the PD group by Pearson's test.

## 3. Results

Epidemiologic and disease characteristics of patients with PD and healthy subjects are shown in Table 1. Mean age in the PD patient group was 67.3 years (range: 54–80) and in the healthy control group 67.2 years (range: 55–79). Intraocular pressure did not differ significantly between groups (*P* = 0.478). The duration of PD ranged from 2 to 19 years, with a mean of 8.5 years since diagnosis. The mean Hoehn-Yahr score was 2.68. The most frequent Hoehn-Yahr stages were stage 2 (56 patients) and stage 3 (41 patients), and mean Schwab-England ADL score was 44.05%. BCVA differed significantly between groups (0.95 in healthy controls, 0.74 in PD patients; *P* < 0.001). Regarding the medications registered in the group of PD patients, there were mainly three different treatment groups: dopamine-enhancers (most common), dopamine agonists, and other drugs (such as anticholinergics and amantadine). We included 10 de novo patients (without medication).

**Figure 2:**
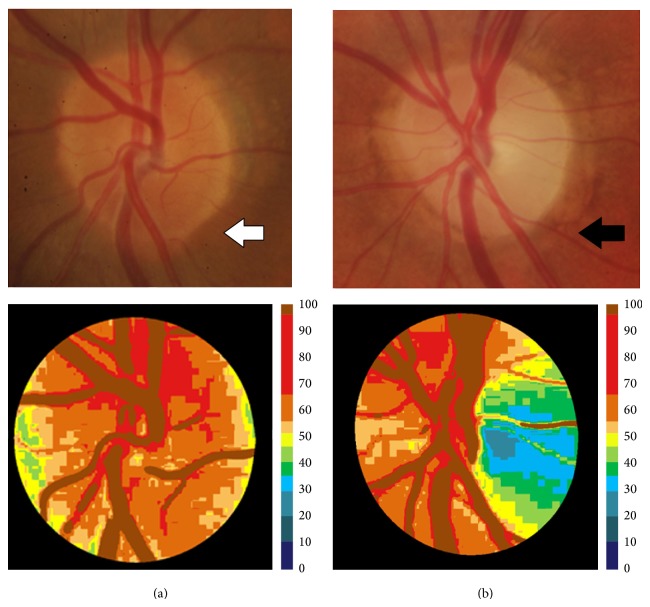
Examples of papillary images in a healthy subject (left column) and a Parkinson's disease patient (right column). Upper images show the color fundus photographs of the optic discs (the white arrow marks the papilla of a healthy subject, and the black arrow marks the papilla of a Parkinson's disease patient). The main parts of a healthy optic nerve can be distinguished in the upper-left image (white arrow): a small “yellow-white” depression is formed in the center of the papilla, which is referred to as the optic cup, to contrast it with the “orange” surrounding neural tissue that is referred to as the neuroretinal rim. Lower images show the corresponding pseudoimages representing the amount of hemoglobin. The colorimetric scale at the right side of the lower images shows the amount of hemoglobin.

Mean Hb percentage and Hb content in sectors 3, 5, 8, 9, 12, 15, 19, and 21 (which mostly correspond with the outer ring of the papilla) calculated by the Laguna ONhE program were significantly lower in PD patients than in healthy controls (*t*-test with Bonferroni correction, *P* < 0.002). The main differences were found in sectors 3, 9, and 12 of the papilla and ranged from 0.77% (in sector 19) to 12.14% (in sector 12) (Table 2).  Figure 2 shows, as an example, the morphology of the papilla and the corresponding pseudoimages indicating the Hb levels provided by Laguna ONhE analysis in a healthy control and a patient with PD.

RNFL measurements evaluated in the study and the significance of the difference between healthy and PD groups are shown in Table 3. Cirrus OCT measurements revealed that RNFL thickness was significantly decreased (*t*-test with Bonferroni correction, *P* < 0.003) in PD patients compared with healthy controls in mean thickness, inferior quadrant thickness, and clock hour sectors 2 and 5 thicknesses. The main difference was detected in the superior quadrant (24.03 *μ*m difference) and inferior quadrant (15.14 *μ*m difference between groups).

The Spectralis OCT revealed a statistically significant reduction of thickness (*t*-test with Bonferroni correction, *P* < 0.006) in the inferior quadrant and the superotemporal RNFL thickness, in the PD group. The main differences were detected in the inferior quadrant (10.14 *μ*m) and superotemporal RNFL thickness (10.02 *μ*m). All structural thicknesses tended to be decreased in patients with PD.

Correlations between the RNFL thickness obtained with Cirrus and Spectralis OCT and the percentage of Hb in the different sectors provided by the Laguna ONhE program were slight. Similarly, correlations between optic nerve Hb levels and Hoehn-Yahr stages in the PD group were slight and not statistically significant.

## 4. Discussion

A study of the rabbit retina revealed dopaminergic neurons, which have subsequently been identified in the inner nuclear layer of the human retina. The principal dopaminergic cell in the retina is an amacrine subtype. These retinal dopaminergic cells are suggested to play a role in the transition from a dark to light-adapted state. PD is associated with not only the death of pigmented dopamine neurons in the substantia nigra, but also a loss of neurons in other areas, such as the dopaminergic amacrine cells, retinal ganglion cells, and cells higher visual areas (e.g., lateral geniculate nucleus, cholinergic nucleus basalis of Meynert, and visual cortex) [21]. This reduction of retinal ganglion cells leads to a corresponding decrease in retinal and RNFL thicknesses that can be detected in PD patients using OCT. Satue et al. and Rohani et al. both demonstrated that Fourier-domain OCTs are valid devices for detecting RNFL atrophy in PD patients [9, 10]. In our study, which included a larger population, we found a reduction in RNFL measurements with the Cirrus and Spectralis devices. A recent study by Roth et al. [22] that included intraretinal layer OCT segmentation reported significant combined outer nuclear and photoreceptor layer thinning in a PD group versus healthy subjects; differences in the RNFL, total macular volume, or other retinal layer thicknesses were not detected. In addition, Ding et al. [23] developed a detailed mathematical model based on raw OCT data to allow for differentiation of the foveae of PD patients from those of healthy controls. These findings indicate the promising future clinical utility of mathematical modeling for evaluating diffuse neurodegenerative conditions.

The Hb amount in each of the 24 sectors of the papilla obtained by the Laguna ONhE analysis was greater in healthy subjects than in PD patients, especially in sectors corresponding with the outer ring of the papilla (Figure 1). The outer ring of the papilla mostly aligns with the neuroretinal rim (where the ganglion cell axons are localized), whereas the medium ring is the transitional area and the inner ring mainly comprises the cup area (which is physiologically pale, because it is the optic disc area without ganglion cell axons and is poorly vascularized). The neuroretinal rim is the papillary area with the greatest vascular supply; therefore, changes in axonal perfusion and/or axonal loss should be observed in this location. The main differences were found in sectors 3, 9, and 12, which correspond with the nasal and inferior regions of the papilla. This is consistent with the results obtained with OCT devices where some of the biggest and significant differences between control and PD groups were also detected in the inferior quadrant (with Cirrus and Spectralis OCT). Correlations between both results (Hb levels and OCT measurements), however, were slight in our population. Although we found no association (loss of ganglion cell axons detected by the OCT would imply a more pale optic disc and thus a lower percentage of Hb detected by Laguna ONhE), long-term studies are needed to obtain statistically reliable results. The lack of a correlation between the two techniques could be due to the different parameters analyzed. The Laguna ONhE software detects changes in the proportion of Hb (and consequently color changes) in different optic disc sectors. This means that the program can detect areas with ischemia and/or a decrease in the number of axons (pale areas). OCT, however, detects and quantifies the RNFL thickness. Therefore, the Laguna ONhE software could detect not only optic disc areas with a decrease in ganglion cells, but also areas with decreased perfusion (prior to death of the neurons). The method used in this study may be applied in all clinical centers because only a good photograph of the optic nerve is required for image processing using this software.

Correlations between optic disc Hb levels and disease severity (assessed by the Hoehn-Yahr stages) were slight and not statistically significant in our group. This result was similar to that of another recent study [22] reporting no association of OCT measures with disease severity and duration. It could be very interesting to investigate the relation between retinal and optic disc changes detected by different digital imaging technologies and the disease severity in PD patients. Large studies with a greater sample size are needed to obtain statistically reliable associations. In another neurodegenerative disease, multiple sclerosis (MS), correlations between retinal layer OCT thicknesses, MS clinical subtype, and other validated clinical measures have been reported, making this a promising approach [24].

A possible limitation of our study could be that the incidence of cataracts in patients with PD is high due to advanced age and surgery may not be performed due to effects of the disease and tremor (although transparent ocular media was an inclusion criterion). The high variability of normal human optic disc morphology (different disc sizes and distribution of the RNFL bundles in the optic nerve head), as well as refractive errors, poor fixation, and eye movements, may affect measurement accuracy. High intraocular pressure, glaucomatous optic nerve head morphology (cup-to-disc ratio of 0.5 or higher), and arcuate nerve fiber bundle visual field defects were the criteria for detection of glaucoma in our study population, so some individuals with low-tension preperimetric glaucoma could have been included in both groups (PD and controls), which may have biased the findings. In addition there was a significant dispersion in Hb values with high standard deviations (SD) (Table 2). This variability could reduce the strength of the conclusions; nevertheless, a clear tendency can be observed in our results. Clinicians should take this limitation into account when using this method, as well as other digital image analysis technologies.

Our research group has already demonstrated the utility of this program in a recent study performed in MS patients, in which we found that the Hb amount in each of the 24 sectors of the papilla was higher in healthy subjects than in MS patients (especially in the temporal sectors, where a greater loss of nerve fibers is observed with OCT), although we did not analyze the correlations between devices [14]. Gonzalez-De la Rosa et al. reported significant linear correlations between mean Hb in sectors 8 and 20 (vertical sectors), with functional and structural tests performed in glaucoma patients, such as OCT (*r* = 0.660 between OCT average thickness and Hb sectors 8–20) [13]. Analysis of Hb levels in optic nerve head photographs using the Laguna ONhE program provided useful and reproducible information about the change in color and perfusion of the papilla secondary to RNFL loss. This method not only detects the paleness of the optic disc (which occurs as a result of axonal loss or optic atrophy), it is able to quantify it.

Longitudinal studies with larger samples are needed to assess the ability of the Laguna ONhE program to provide a new biomarker of neurodegeneration in PD and other neurodegenerative diseases. Our findings suggest that this program has an important clinical application, but these findings should be confirmed and extended for other ophthalmologic diseases causing axonal loss in the optic disc.

## 5. Conclusions

Our findings demonstrate that Hb content is lowest in some fields of the optic nerve head in PD patients compared with age-matched healthy subjects. This supposes that optic nerve in PD patients is paler than in control group, and we can quantify it by using Laguna ONhE software. Our study suggests that this simple, inexpensive, and noninvasive technique may detect papillary paleness in PD patients and it could suppose axonal loss in the optic nerve head.

## Figures and Tables

**Table 1:** Epidemiologic and disease characteristics of 155 patients with Parkinson's disease and 91 healthy subjects included in the study and statistical significance of comparison between both groups (*P*).

	Healthy subjects (*n* = 91)	Patients with Parkinson's disease (*n* = 155)	*P* ^*^
Age (years) : mean (range)	67.21 (55–79)	67.33 (54–80)	—
Male : female (% men)	54 : 37 (59%)	92 : 65 (59%)	—
Intraocular pressure (SD)	16.67 (1.5)	16.56 (1.71)	0.478
BCVA (Snellen scale); mean (SD)	0.95 (0.08)	0.74 (0.18)	<0.001
Disease duration (yrs); mean (SD)	—	8.52 (2.10)	—
Hoehn and Yahr Score	—	2.68 (0.82)	—
Schwab-England activity daily living scale	—	44.05 (15.97)	—

BCVA: best-corrected visual acuity; SD: standard deviation. ^*^Significant difference (*P* < 0.05) between normal subjects and Parkinson's disease patients for each population.

**Table 2:** Mean and standard deviation in parenthesis of hemoglobin's percentage analysing optic disc photographs with Laguna ONhE program (optic nerve head hemoglobin) of Parkinson's disease patients and healthy subjects and statistical significance of the comparison between both groups (*P*).

	Healthy subjects	Parkinson's patients	*P* ^*^
Mean Hb	67 (11)	58 (19)	**0.001**
Hb 1	74 (16)	70 (16)	0.251
Hb 2	73 (14)	64 (16)	0.010
Hb 3	68 (12)	56 (14)	**<0.001**
Hb 4	71 (15)	65 (18)	0.096
Hb 5	74 (13)	64 (15)	**0.001**
Hb 6	68 (12)	58 (24)	0.002
Hb 7	64 (17)	60 (16)	0.316
Hb 8	71 (11)	60 (14)	**<0.001**
Hb 9	69 (11)	56 (15)	**<0.001**
Hb 10	57 (16)	54 (12)	0.360
Hb 11	58 (17)	49 (12)	0.011
Hb 12	61 (13)	49 (12)	**<0.001**
Hb 13	57 (17)	52 (16)	0.247
Hb 14	54 (17)	47 (16)	0.032
Hb 15	58 (15)	46 (12)	**0.001**
Hb 16	60 (17)	57 (15)	0.428
Hb 17	64 (17)	57 (14)	0.036
Hb 18	67 (14)	57 (19)	0.002
Hb 19	67 (19)	66 (19)	0.835
Hb 20	77 (14)	69 (16)	0.005
Hb 21	76 (11)	65 (18)	**0.001**
Hb 22	73 (17)	71 (19)	0.724
Hb 23	84 (13)	71 (19)	0.005
Hb 24	75 (12)	64 (17)	**0.001**

Hb: hemoglobin. ^*^Significant difference (*P* < 0.002) in Student's *t*-test with Bonferroni correction for multiple comparison between healthy subjects and PD patients.

**Table 3:** Mean and standard deviation of retinal nerve fiber layer thicknesses obtained with the Cirrus and Spectralis optical coherence tomography devices in healthy controls and patients with Parkinson's disease.

	Parameters	Healthy controls	PD patients	*P* ^*^
RNFL Cirrus measurements	Mean thickness	105 (10)	93 (10)	**<0.001**
	Superior quadrant	139 (19)	115 (17)	0.007
	Nasal quadrant	80 (11)	73 (13)	0.005
	Inferior quadrant	134 (21)	119 (18)	**<0.001**
	Temporal quadrant	66 (11)	65 (13)	0.564
	Hour sector 1	118 (23)	102 (15)	0.004
	Hour sector 2	101 (19)	86 (19)	**0.002**
	Hour sector 3	64 (10)	56 (11)	0.199
	Hour sector 4	75 (13)	62 (14)	0.019
	Hour sector 5	117 (22)	94 (16)	**<0.001**
	Hour sector 6	149 (24)	130 (20)	0.003
	Hour sector 7	137 (26)	129 (24)	0.112
	Hour sector 8	70 (15)	68 (14)	0.290
	Hour sector 9	60 (10)	55 (10)	0.277
	Hour sector 10	78 (15)	75 (14)	0.306
	Hour sector 11	136 (23)	130 (22)	0.018
	Hour sector 12	126 (22)	119 (25)	0.183

RNFL Spectralis measurements	Mean thickness	100 (10)	93 (10)	0.030
	Superior quadrant	123 (13)	115 (26)	0.019
	Nasal quadrant	73 (13)	71 (26)	0.667
	Inferior quadrant	133 (20)	122 (27)	**0.005**
	Temporal quadrant	71 (11)	64 (23)	0.023
	Superior nasal	126 (17)	122 (18)	0.091
	Inferior nasal	141 (18)	138 (18)	0.124
	Inferior temporal	108 (21)	99 (18)	0.007
	Superior temporal	113 (15)	103 (19)	**0.002**

PD: Parkinson's disease; RNFL: retinal nerve fiber layer. ^*^Significant difference (*P* < 0.003 for Cirrus measurements and *P* < 0.006 for Spectralis measurements) in Student's *t*-test with Bonferroni correction between healthy subjects and PD patients.
